# Relative Pose Based Redundancy Removal: Collaborative RGB-D Data Transmission in Mobile Visual Sensor Networks

**DOI:** 10.3390/s18082430

**Published:** 2018-07-26

**Authors:** Xiaoqin Wang, Y. Ahmet Şekercioğlu, Tom Drummond, Vincent Frémont, Enrico Natalizio, Isabelle Fantoni

**Affiliations:** 1 ARC Centre of Excellence for Robotic Vision, Monash University, Victoria 3800, Australia; xiaoqin.wang@monash.edu (X.W.); tom.drummond@monash.edu (T.D.); 2 Université de Technologie de Compiègne, Sorbonne Universités, CNRS, UMR 7253 Heudiasyc-CS 60 319, 60203 Compiègne, France; vincent.fremont@utc.fr (V.F.); enrico.natalizio@utc.fr (E.N.); 3 Ecole Centrale de Nantes, CNRS, UMR 6004 LS2N, 44300 Nantes, France; isabelle.fantoni@ls2n.fr

**Keywords:** RGB-D sensors, 3D mapping, visual sensors, robotic vision, collaborative coding, relative pose estimation

## Abstract

In this paper, the *Relative Pose based Redundancy Removal* (RPRR) scheme is presented, which has been designed for mobile RGB-D sensor networks operating under bandwidth-constrained operational scenarios. The scheme considers a multiview scenario in which pairs of sensors observe the same scene from different viewpoints, and detect the redundant visual and depth information to prevent their transmission leading to a significant improvement in wireless channel usage efficiency and power savings. We envisage applications in which the environment is static, and rapid 3D mapping of an enclosed area of interest is required, such as disaster recovery and support operations after earthquakes or industrial accidents. Experimental results show that wireless channel utilization is improved by 250% and battery consumption is halved when the RPRR scheme is used instead of sending the sensor images independently.

## 1. Introduction

Visual sensor networks (VSNs) allow the capture, processing, and transmission of per-pixel color information from a variety of viewpoints. The inclusion of low-cost compact RGB-D sensors, such as Microsoft Kinect [1], Asus Xtion [2] and Intel RealSense ZR300 [3], makes VSNs able to collect depth data as well.

**Figure 1:**
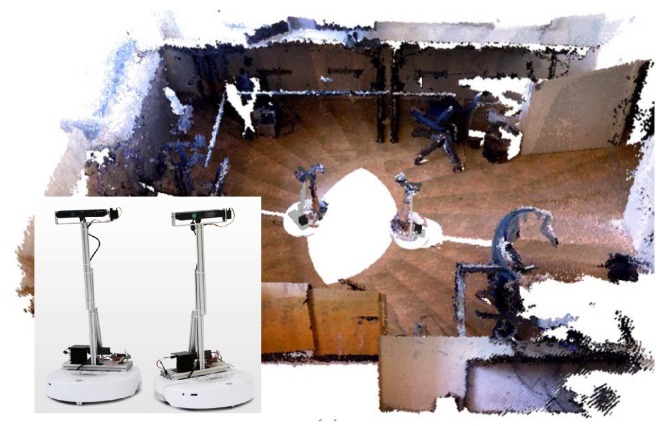
An example of 3D indoor mapping with two simultaneously operating mobile RGB-D sensor platforms [4].

RGB-D sensor-equipped VSNs can significantly enhance the performance of conventional applications such as immersive telepresence or mapping [4,5,6,7], environment surveillance [8,9], or object recognition and tracking [10,11,12] as well as opening the possibilities for new and innovative applications like hand gesture recognition [13], indoor positioning systems [14] and indoor relocalization [15]. The value of VSN applications becomes even more important, especially in places inaccessible to humans, such as supporting search and rescue operations after earthquakes, industrial or nuclear accidents. Indeed, examples of mapping (especially indoors) with networked mobile RGB-D sensors have started to appear in the research literature (Figure 1).

RGB-D sensors generate visual and depth data inevitably in huge quantities. The data volume will be even larger when multiple camera sensors observe the same scene from different viewpoints and exchange/gather their measurements to better understand the environment. As the sensors will most likely be communicating in ad hoc networking configurations, communication bandwidth will be at a premium, and will be error-prone and not suitable for continuous data delivery in large quantities. Moreover, wireless transceivers consume a significant portion of the available battery power [16], and capacity limitation of on-board power sources should also be considered. Consequently, transmission of visual and depth information in resource-constrained VSN nodes must be carefully controlled and minimized as much as possible.

As the same scenery may be observed by multiple sensors (like the example shown in Figure 1), collected images will inevitably contain a significant amount of correlated information, and transmission load will be unnecessarily high if all the captured data are sent. In this paper, we focus on this issue and present a novel approach to the development of a comprehensive solution for minimizing the transmission of redundant RGB-D data in VSNs. Our framework, called *Relative Pose based Redundancy Removal* (RPRR), efficiently removes the redundant information captured by each sensor before transmission. We designed the RPRR framework particularly for RGB-D sensor-equipped VSNs, which eventually will need to work in situations with severely limited communication bandwidth. The scheme operates fully on board.

In the RPRR framework, the characteristics of depth images, captured simultaneously with color data, are used to achieve the desired efficiency. Instead of using a centralized image registration technique [17,18], which requires one node to have full knowledge of the images captured by the others to determine the correlations, we propose a new approach based on relative pose estimation between pairs of RGB-D sensors and the 3D image warping technique [19]. The method we propose locally determines the color and depth information, which can only be seen by one sensor but not the others. Consequently, each sensor is required to transmit only the uncorrelated information to the remote station. In order to further reduce the amount of information before transmission, we apply a conventional coding scheme based on the discrete wavelet transform [20] with progressive coding features for color images, and a novel lossless differential entropy coding scheme for depth images (this algorithm was published in an earlier paper [21]). In addition, at the remote monitoring station, to deal with the artifacts that could occur in the reconstructed images due to the undersampling problem [22], we use our post-processing algorithms.

Early results of this work were presented in [23], and in this paper we

1. Add detailed theoretical refinements, practical implementation and experimental performance evaluation of the cooperative relative pose estimation algorithm [24] (Section 3.2),
2. Extend the theoretical development and practical implementation of the RPRR scheme for minimizing the transmission of redundant RGB-D data collected over multiple sensors with large pose differences (Section 3.3),
3. Describe the lightweight crack and ghost artifacts removal algorithms as a solution to the undersampling problem (Section 3.5), and
4. Include detailed experimental evaluation of wireless channel capacity utilization and energy consumption (Section 4.2).

In the following sections of the paper, after a discussion of the related work, we present the details of the RPRR framework in Section 3, and experimental results and their analysis can be found in Section 4, followed by our concluding remarks.

## 2. Related Work

A number of solutions exist in the research literature that intend to remove or minimize the correlated data for transmission in VSNs. They can be broadly classified into three groups:

- Optimal camera selection,
- Collaborative compression and transmission, and
- Distributed source coding.

The optimal camera selection algorithms [25,26,27,28,29] attempt to group the camera sensors with overlapping fields-of-view (FoVs) into clusters and only activate the sensor that can capture the image with the highest number of feature points. The pioneering work presented in [29] demonstrated that a correlation-based algorithm can be designed for selecting a suitable group of cameras communicating toward a sink so that the amount of information from the selected cameras can be maximized. Based on this work, in [28], the concept of “common sensed area” was proposed between two views to measure the efficiency of multiview video coding techniques and reduce the amount of information transmitted in VSNs. These algorithms operate under the assumption that the images captured by a small number of camera sensors in one cluster are good enough to represent the information of the scene/object. In these approaches, the location and orientation of the camera sensors are used to establish clusters, and a variety of existing feature detection algorithms [30,31] or place recognition approaches [15,32] are used to determine the similarity between captured images in each cluster. However, the occlusions in FoVs may cause significant differences between the images captured by cameras with very similar sensing directions. Therefore, the assumption is not realistic and this kind of approach is not applicable in many situations.

The collaborative compression and transmission methods [33,34,35,36,37] jointly encode the captured multi-view images. The spatial correlation is explored and removed at encoders by image registration algorithms. Only the uncorrelated visual content is delivered in the network after being jointly encoded by some recent coding techniques (e.g., Multiview Video Coding (MVC) [38,39]) and compressive sensing approaches [40,41]. However, at least one node in the network is required to have the full set of images captured by the other sensors in order to perform image registration. This means that the redundant information cannot be removed completely and still needs to be transmitted at least once. Moreover, as color images do not contain a full 3D representation of a scene, these methods introduce distortions and errors when the relative poses (location and orientation) between sensors are not pure rotation or translation, or the scenes have complex geometrical structures and occlusions.

The distributed source coding (DSC) algorithms [42,43,44,45,46] are other promising approaches that can be used to reduce the redundant data in multiview VSN scenarios. Each DSC encoder operates independently, but, at the same time, relies on joint decoding operations at the sink (remote monitoring station). The advantage of these approaches is that the camera sensors do not need to directly communicate the captured visual information with others in the network. Furthermore, these algorithms shift the computational complexity from the sensor nodes to the remote monitoring station, which fits the needs of VSNs well. However, the side information must be predicted as accurately as possible and the correlation structure should be able to be identified at the decoder side (remote monitoring station), without an accurate knowledge of the network topology and the poses of the sensors. These are the main disadvantages that prevent DSC algorithms from being widely implemented. A detailed discussion on multi-view image compression and transmission schemes in VSNs is presented in [47].

The algorithms mentioned above focus only on color (RGB) data. Just a few studies have been reported [4,48,49] that use RGB-D sensors in VSNs, as their use in networked robotics scenarios has not yet become widespread. Consequently, our extensive review of the research literature has not identified any earlier studies that attempt to develop an efficient coding system that aims to maximize the bandwidth usage and minimize the energy consumption for RGB-D equipped VSNs.

## 3. Relative Pose Based Redundancy Removal (RPRR) Framework

### 3.1. Overview

In a mobile VSN tasked with mapping a region using RGB-D sensors, it is highly possible that multiple sensors will observe the same scene from different viewpoints. Because of this, scenery captured by the sensors with overlapping FoVs will have a significant level of correlated and redundant information. Here, our goal is to efficiently extract and encode the uncorrelated RGB-D information, and avoid transmitting the same surface geometry and color information repeatedly.

Consider the two sensors, *a* and *b*, of this VSN with overlapping FoVs. Let $Z_{a}$ and $Z_{b}$ denote a pair of depth images returned by these sensors, and $C_{a}$ and $C_{b}$ are the corresponding color images. In the encoding procedure, we first estimate the location and orientation of one sensor relative to the other. Then, correlated and redundant information in color and depth images are identified to minimize unnecessary data transmissions to the central monitoring station. To achieve this, by using the relative pose information, sensor *a* computes a prediction of $Z_{b}$ to determine the depth and color information that exists only in $Z_{b}$ but not in $Z_{a}$. Then, it informs sensor *b* to send only the uncorrelated depth and corresponding color information in $Z_{b}$ and $C_{b}$. To further improve the wireless channel capacity usage, depth image data is compressed with our own *Differential Huffman Coding with Multiple Lookup Tables (DHC-M)* method [21], and color images are compressed with Progressive Graphics File (PGF) scheme [50] prior to their transmission.

At the remote monitoring station, to improve the image quality, we apply algorithms for removal of the visual artifacts that may be introduced during the image reconstruction process.

**Figure 2:**
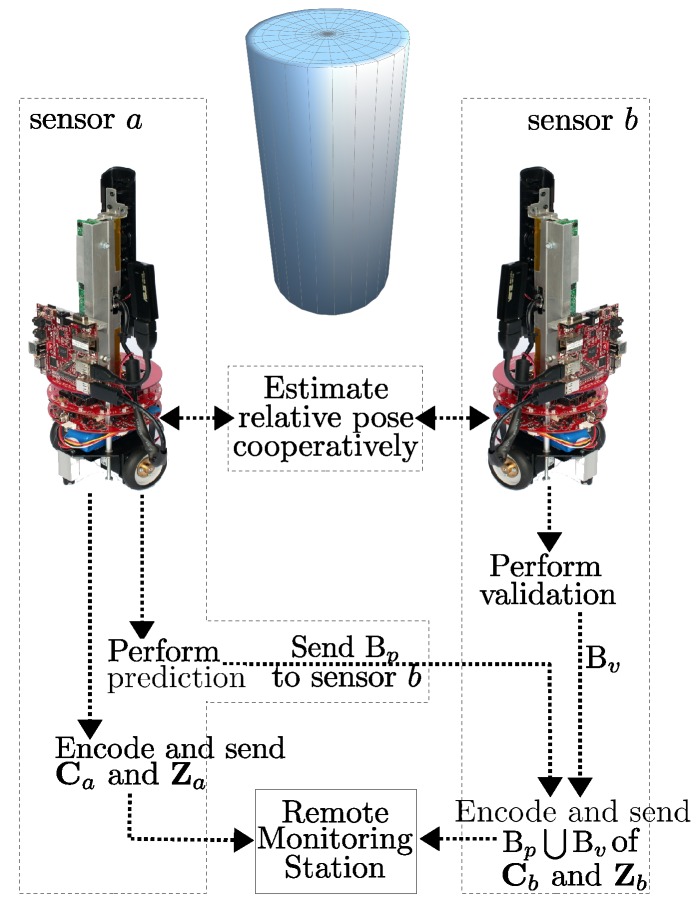
Operational overview of the RPRR framework. The sensors first cooperatively estimate their relative poses by using the algorithm shown in Figure 4, then, after identifying the non-overlapping image blocks, send only the non-redundant visual information to the remote monitoring station. Here, $C_{a}$, $C_{b}$ and $Z_{a}$, $Z_{b}$ are color and depth images obtained by sensors *a* and *b*, $B_{p}$ is the set of image block coordinates that can only be observed by sensor *b*, and $B_{v}$ is the set of image block coordinates covering the regions that sensor *a* incorrectly estimates as visible by sensor *b*.

A high-level view of the operation of the system is shown in Figure 2. A detailed explanation of each step is provided in the following sections.

### 3.2. Relative Pose Estimation

As an RGB-D sensor can provide a continuous measurement of the 3D structure of the environment, the relative pose between two RGB-D sensors can be estimated through explicit matching of surface geometries in the overlapping regions within their FoVs. A variety of algorithms have been proposed to determine whether multiple cameras are looking at the same scene, such as vision-based [27,51] or geometry-based [29,52] methods. Here, we assume that the sensors use one of these approaches to detect whether they are observing the same scene. Afterwards, as explained below, with our relative pose estimation algorithm, the sensors accurately estimate their relative position and orientation (relative pose).

The relative pose between the RGB-D sensors *a* and *b* can be represented by a transformation matrix:

$$\begin{array}{c} M_{ab}=\left[\begin{array}{cccc} \; & R & \; & t\\ 0 & 0 & 0 & 1 \end{array}\right] \end{array}$$

in SE(3) [53], where R is a 3×3 rotation matrix and t is a 3×1 translation vector. The transformation matrix $M_{\mathrm{ab}}$ represents the six degrees of freedom (6DoF) motion model, which not only describes the relative pose between two sensors and also the transformation of the structure between the depth images captured by both sensors.

The transformation matrix $M_{ab}$ can be estimated by matching the surface geometries captured by two sensors. Taking advantage of the depth image characteristics, the depth pixels in a frame captured by sensor *b* can be mapped to a frame captured by sensor *a*. Consider the vector $p_{e}={\left[x\;y\;z\;1\right]}^{T}$ which represents a real world point in Euclidean space by using homogeneous coordinates. Given the following intrinsic parameters of an RGB-D sensor:

- principal point coordinates $(i_{c}$, $j_{c})$ and
- focal length of the camera $(f_{x}$, $f_{y})$,

$p_{e}$ can be estimated from the corresponding pixel in a depth image by using the pinhole camera model as

$$\frac{1}{z}p_{e}=\frac{1}{z}{\left[\begin{array}{cccc} x & y & z & 1 \end{array}\right]}^{T}={\left[\begin{array}{cccc} \frac{i-i_{c}}{f_{x}} & \frac{j-j_{c}}{f_{y}} & 1 & \frac{1}{z} \end{array}\right]}^{T},$$

where (i,j) denotes the pixel coordinates of the projection of this real world point in the depth image, and *z* is the corresponding depth value reported by the camera.

In the discussion that follows, we assume that $p_{e}$ can be observed by both mobile RGB-D sensors *a* and *b*, and the projections of $p_{e}$ are located at pixel coordinates $\left(i_{a},j_{a}\right)$ and $\left(i_{b},j_{b}\right)$ on the depth images $Z_{a}$ and $Z_{b}$, respectively. Under the assumption that the world coordinate system is equal to the mobile sensor coordinate system, and the intrinsic parameters of both sensors are identical, the depth pixel (projection) at $\left(i_{a},j_{a}\right)$ in $Z_{a}$ can establish a relationship between the depth pixel at $\left(i_{b},j_{b}\right)$ in $Z_{b}$ as

(1) $${\left[\begin{array}{cccc} \frac{i_{b}-i_{c}}{f_{x}}\; & \;\frac{j_{b}-j_{c}}{f_{y}}\; & \;1\; & \;\frac{1}{z_{b}} \end{array}\right]}^{T}\;\;=M_{ab}\;{\left[\begin{array}{cccc} \frac{i_{a}-i_{c}}{f_{x}}\; & \;\frac{j_{a}-j_{c}}{f_{y}}\; & \;1\; & \;\frac{1}{z_{a}} \end{array}\right]}^{T}$$

and, to simplify the equation, by doing some rudimentary algebraic substitutions, we obtain

$$\begin{array}{c} {\left[u_{b}\;v_{b}\;1\;q_{b}\right]}^{T}=M_{ab}{\left[u_{a}\;v_{a}\;1\;q_{a}\right]}^{T} \end{array}$$

in inverse depth coordinates.

We now need to estimate $M_{ab}$. To accomplish an accurate estimate of $M_{ab}$, we have developed an Iterative Closest Point (ICP) algorithm which operates in a distributed fashion by using the explicit registration of surface geometries extracted from the depth frames captured by two sensors [24]. It delivers robust results especially in circumstances with heavy occlusion. In our distributed algorithm, the registration problem is approached by iteratively minimizing a cost function whose error metric is defined based on the bidirectional point-to-plane geometrical relationship as explained in the following paragraphs.

**Figure 3:**
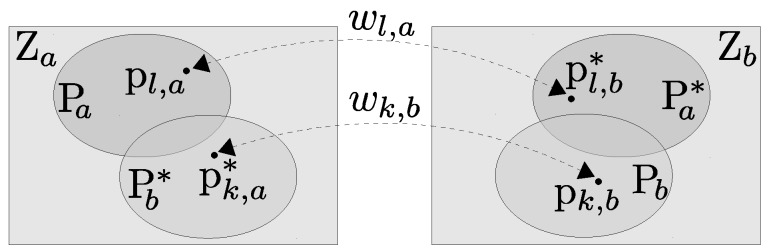
Two sets of points ($P_{\;a}\subset Z_{a}$ and $P_{b}\subset Z_{b}$) sampled from the depth images $Z_{a}$ and $Z_{b}$, and their corresponding point sets $P_{\;a}^{*}$ and $P_{\;b}^{*}$. $P_{\;a}$ and $P_{b}$ have $N_{a}$ and $N_{b}$ number of elements, respectively. For finding the point sets, the project-and-walk method is used with a neighborhood size of 7 × 7 pixels based on the nearest neighbor criteria as proposed in [54].

Let $P_{\;a}=\left\{p_{l,a},l=1,2,\cdots ,N_{a}\right\}$ and $P_{b}=\left\{p_{k,b},k=1,2,\cdots ,N_{b}\right\}$ denote two sets of measurements sampled from $Z_{a}$ and $Z_{b}$. Let us assume that the correspondences for $N=N_{a}+N_{b}$ pairs (A typical depth image may have hundreds of thousands of points, therefore running algorithms on the full point cloud is computationally expensive. In order to alleviate this problem, a commonly used method is to subsample the data for speeding up the operation with the cost of reduced accuracy. This is a fundamental trade-off of ICP performance: registration by using dense point clouds yields a more accurate alignment, however it needs longer processing time to complete. On the other hand, a subsampled point cloud results in lower accuracy, but requires a significantly shorter processing time. Thus, for the best ICP (and its variants) performance, striking a balance between accuracy and processing time is required by considering the timing requirements for obtaining results and the computational resources available. Considering these, after conducting a series of experiments on our sensor platforms, we have chosen $N_{a}=N_{b}=250$.) of points $\left(p_{l,a}\;\;↔\;p_{l,b}^{*}\right)$ and $\left(p_{k,b}\;\;↔\;p_{k,a}^{*}\right)$ are established to form the sets $P_{\;a}^{*}$ and $P_{b}^{*}$. Here, $p_{l,b}^{*}\in P_{\;a}^{*}$ (where $P_{\;a}^{*}\subset Z_{b}$) is the corresponding point of $p_{l,a}$, and $p_{k,a}^{*}\in P_{b}^{*}$ (where $P_{b}^{*}\subset Z_{a}$) is the corresponding point of $p_{k,b}$ (see Figure 3). Then, the transformation matrix $M_{ab}$ can be estimated by minimizing the bidirectional point-to-plane error metric C, expressed in normal least squares form as

(2) $$C=\sum_{l=1}^{N_{a}}{\left[w_{l,a}{\left(M_{ab}p_{l,a}-p_{l,b}^{*}\right)}^{T}{\vec{\;\;n}}_{l,b}^{*}\right]}^{2}+\sum_{k=1}^{N_{b}}{\left[w_{k,b}{\left(M_{ab}^{-1}p_{k,a}^{*}-p_{k,b}\right)}^{T}{\vec{\;\;n}}_{k,b}\right]}^{2},$$

where $w_{l,a}$ and $w_{k,b}$ are the weight parameters for the correspondences established in opposite directions between the pairs,

(3) $$\begin{array}{cc} {\vec{\;\;n}}_{l,b}^{*}\;= & {\left[\begin{array}{cccc} \beta_{l,b}^{*} & \gamma_{l,b}^{*} & \delta_{l,b}^{*} & 0 \end{array}\right]}^{T} \end{array}$$

and

(4) $$\begin{array}{cc} {\vec{\;\;n}}_{k,b}= & {\left[\begin{array}{cccc} \beta_{k,b} & \gamma_{k,b} & \delta_{k,b} & 0 \end{array}\right]}^{T}, \end{array}$$

are the surface normals at the points $p_{l,b}^{*}$ and $p_{k,b}$. The cost function presented in Equation (2) consists of two parts:

- the sum of squared distances from $Z_{a}$ to $Z_{b}$, and
- the sum of squared distances from $Z_{b}$ to $Z_{a}$.

**Figure 4:**
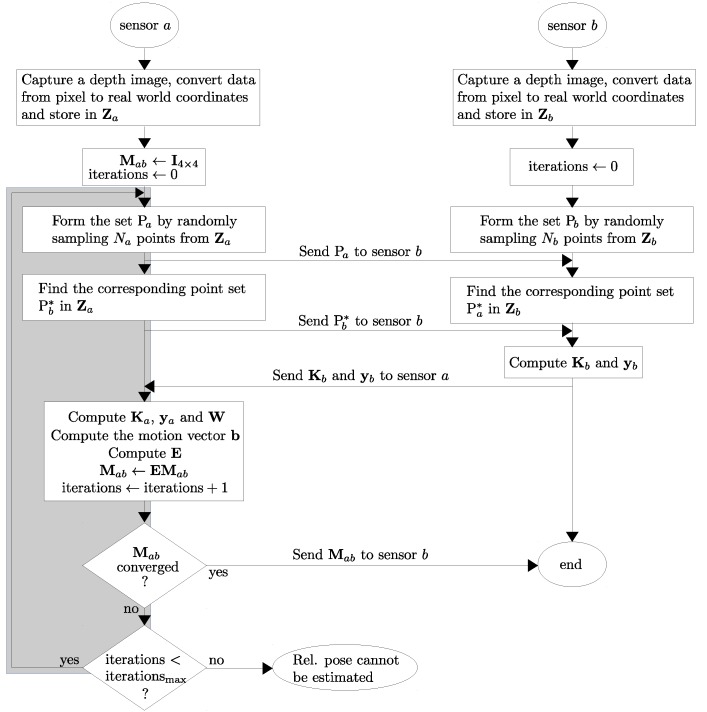
Operation of the cooperative relative pose estimation algorithm. The algorithm is distributed over two sensors, and operates iteratively (denoted in gray) until it converges or maximum number of iterations is reached. We have used the convergence criterion presented in [55], and ${\mathrm{iterations}}_{\max}$ is set as 50 (see Section 4.1).

The estimation of $M_{ab}$ can be done by iteratively re-weighting the least squares operation in an ICP framework. Based on this principle, we have created the distributed algorithm which has two complementary components running concurrently on sensors *a* and *b* as shown in Figure 4.

On sensor *a*, in the first iteration, $M_{ab}$ is initialized as the identity matrix. Afterwards, in this coarse-to-fine algorithm, by using the information sent by sensor *b*, each iteration generates an update E to the sensor’s pose, which modifies the transformation matrix $M_{ab}$. E takes the same form as $M_{ab}$ and can be parameterized by a six-dimensional motion vector having the elements $\alpha_{1},\alpha_{2},\cdots ,\alpha_{6}$ via the exponential map and their corresponding group generator matrices $G_{1},G_{2},\cdots ,G_{6}$ as

(5) $$E=\exp\;\;\left(\sum_{j=1}^{6}\alpha_{j}G_{j}\right),$$

where

$$\begin{array}{ccc} G_{1}=[\begin{array}{cccc} 0 & 0 & 0 & 1\\ 0 & 0 & 0 & 0\\ 0 & 0 & 0 & 0\\ 0 & 0 & 0 & 0 \end{array}] & G_{2}=[\begin{array}{cccc} 0 & 0 & 0 & 0\\ 0 & 0 & 0 & 1\\ 0 & 0 & 0 & 0\\ 0 & 0 & 0 & 0 \end{array}] & G_{3}=[\begin{array}{cccc} 0 & 0 & 0 & 0\\ 0 & 0 & 0 & 0\\ 0 & 0 & 0 & 1\\ 0 & 0 & 0 & 0 \end{array}]\\ G_{4}=[\begin{array}{cccc} 0 & 0 & 0 & 0\\ 0 & 0 & -1 & 0\\ 0 & 0 & 0 & 0\\ 0 & 0 & 0 & 0 \end{array}] & G_{5}=[\begin{array}{cccc} 0 & 0 & 1 & 0\\ 0 & 0 & 0 & 0\\ -1 & 0 & 0 & 0\\ 0 & 0 & 0 & 0 \end{array}] & G_{6}=[\begin{array}{cccc} 0 & -1 & 0 & 0\\ 1 & 0 & 0 & 0\\ 0 & 0 & 0 & 0\\ 0 & 0 & 0 & 0 \end{array}] \end{array}$$

Here, $G_{1}$, $G_{2}$ and $G_{3}$ are the generators of translations in *x*, *y* and *z* directions, while $G_{4}$, $G_{5}$ and $G_{6}$ are rotations about *x*, *y* and *z* axes, respectively. For details, please refer to [56,57]. The task then becomes finding the elements of the six-dimensional motion vector

$$b={\left[\begin{array}{cccccc} \alpha_{1} & \alpha_{2} & \alpha_{3} & \alpha_{4} & \alpha_{5} & \alpha_{6} \end{array}\right]}^{T}$$

that describe the relative pose. By determining the partial derivatives of $u_{b}$, $v_{b}$ and $q_{b}$ with respect to the unknown elements of b, the Jacobian matrix for each established corresponding point pair can be obtained as

(6) $$\begin{array}{c} J=\left[\begin{array}{cccccc} q_{a} & 0 & -u_{a}q_{a} & -u_{a}v_{a} & 1+u_{a}^{2} & -v_{a}\\ 0 & q_{a} & -v_{a}q_{a} & -1-v_{a}^{2} & v_{a}u_{a} & u_{a}\\ 0 & 0 & -q_{a}^{2} & -v_{a}q_{a} & u_{a}q_{a} & 0 \end{array}\right]. \end{array}$$

The six-dimensional motion vector b, which minimizes Equation (2), is then determined iteratively by the least squares solution

(7) $$\begin{array}{c} b={\left(K^{T}WK\right)}^{-1}K^{T}Wy, \end{array}$$

in which

(8) $$K=\left[\begin{array}{c} K_{b}\\ K_{a} \end{array}\right],$$

where

(9) $$\begin{array}{cc} K_{b}=\left[\begin{array}{c} {\left({\vec{n}}_{1,b}^{*\prime }\right)}^{T}J_{1,a}^{}\\ \vdots \;\vdots \\ {\left({\vec{n}}_{l,b}^{*\prime }\right)}^{T}J_{l,a}^{}\\ \vdots \;\vdots \\ {\left({\vec{n}}_{N_{a},b}^{*\prime }\right)}^{T}J_{N_{a},a}^{} \end{array}\right] & K_{a}=\left[\begin{array}{c} {\left({\vec{n}}_{1,b}^{\prime }\right)}^{T}J_{1,a}^{*}\\ \vdots \;\vdots \\ {\left({\vec{n}}_{k,b}^{\prime }\right)}^{T}J_{k,a}^{*}\\ \vdots \;\vdots \\ {\left({\vec{n}}_{N_{b},b}^{\prime }\right)}^{T}J_{N_{b},a}^{*} \end{array}\right], \end{array}$$

${\vec{\;\;n}}_{l,b}^{*\prime }={\left[\begin{array}{ccc} \beta_{l,b} & \gamma_{l,b} & \delta_{l,b} \end{array}\right]}^{T}$, ${\vec{\;\;n}}_{k,b}^{\prime }={\left[\begin{array}{ccc} \beta_{k,b} & \gamma_{k,b} & \delta_{k,b} \end{array}\right]}^{T}$ are the surface normals at the points $p_{l,b}^{*}\in P_{\;a}^{*}$ and $p_{k,b}^{}\in P_{b}^{}$ expressed in a slightly different form than in Equations (3) and (4), and

(10) $$\begin{array}{c} J_{l,a}^{}=\left[\begin{array}{cccccc} q_{l,a} & 0 & -u_{l,a}q_{l,a} & -u_{l,a}v_{l,a} & 1+u_{l,a}^{2} & -v_{l,a}\\ 0 & q_{l,a} & -v_{l,a}q_{l,a} & -1-v_{l,a}^{2} & v_{l,a}u_{l,a} & u_{l,a}\\ 0 & 0 & -q_{l,a}^{2} & -v_{l,a}q_{l,a} & u_{l,a}q_{l,a} & 0 \end{array}\right] \end{array}$$

is the associated Jacobian matrix calculated over the corresponding point $p_{l,a}\in P_{a}$ (see Figure 3). Similarly,

(11) $$\begin{array}{c} J_{k,a}^{*}=\left[\begin{array}{cccccc} q_{k,a}^{*} & 0 & -u_{k,a}^{*}q_{k,a}^{*} & -u_{k,a}^{*}v_{k,a}^{*} & 1+u_{k,a}^{*2} & -v_{k,a}^{*}\\ 0 & q_{k,a}^{*} & -v_{k,a}^{*}q_{k,a}^{*} & -1-v_{k,a}^{*2} & v_{k,a}^{*}u_{k,a}^{*} & u_{k,a}^{*}\\ 0 & 0 & -q_{k,a}^{*2} & -v_{k,a}^{*}q_{k,a}^{*} & u_{k,a}^{*}q_{k,a}^{*} & 0 \end{array}\right] \end{array}$$

is the associated Jacobian matrix calculated over the corresponding point $p_{k,a}^{*}\in P_{\;b}^{*}$, and

(12) $$y=\left[\begin{array}{c} y_{b}\\ y_{a} \end{array}\right],$$

where

(13) $$\begin{array}{cc} y_{b}=\left[\begin{array}{c} -{\left(p_{1,a}-p_{1,b}^{*}\right)}^{T}{\vec{\;\;n}}_{1,b}^{*}\\ \vdots \\ -{\left(p_{N_{a},a}^{}-p_{N_{a},b}^{*}\right)}^{T}{\vec{\;\;n}}_{N_{a},b}^{*} \end{array}\right] & y_{a}=\left[\begin{array}{c} -{\left(p_{1,a}^{*}-p_{1,b}^{}\right)}^{T}{\vec{\;\;n}}_{1,b}^{}\\ \vdots \\ -{\left(p_{N_{b},a}^{*}-p_{N_{b},b}^{}\right)}^{T}{\vec{\;\;n}}_{N_{b},b}^{} \end{array}\right]. \end{array}$$

In addition,

(14) $$W=\left[\begin{array}{ccc} w_{1,1} & \cdots & 0\\ \vdots & \ddots & \vdots \\ 0 & \cdots & w_{\;N,N}^{} \end{array}\right]$$

contains the weightings for the bidirectional point-to-plane correspondences. As reported in [58], different weighting functions lead to various probability distributions. Based on our experiments, we have found that the asymmetric weighting function

(15) $$\begin{array}{c} w_{a,b}=\left\{\begin{array}{cc} c/[c+\left(z_{a}-z_{b}\right)], & \;\mathrm{if}\;z_{b}\leq z_{a}\;,\\ c/[c+{\left(z_{a}-z_{b}\right)}^{2}], & \;\mathrm{otherwise} \end{array}\right. \end{array}$$

yields satisfactory results. Here, $z_{a}$ and $z_{b}$ are the depth values of corresponding points in two depth images, and *c* is the mean of differences between the depth values of all corresponding points. An extended discussion of the weighting function can be found in our paper [24].

To detect the convergence of our algorithm, we use the thresholds for the ICP framework presented in [55]. Once the algorithm converges, the registration is considered completed and $M_{ab}$ is used for the elimination of the redundant data in transmissions as explained in Section 3.3.

The sensors exchange very small amounts of information by using this algorithm making the process very bandwidth-efficient fitting the requirements of VSNs. We present an in-depth analysis of message exchange complexity in Section 4.1.

### 3.3. Identification of Redundant Regions in Images

#### 3.3.1. Prediction

Sensor *a*, by using the relative pose information $M_{ab}$, can now apply Equation (1) on each pixel in $Z_{a}$ to create a predicted a depth image $Z_{b}^{*}$, which is virtually captured from sensor *b*’s viewpoint. In this process, though, it could happen that two or more different depth pixels are warped into the same pixel coordinates in $Z_{b}^{*}$. This over-sampling issue could occur because some 3D world points are occluded by the other ones at the new viewpoint. In order to solve this problem, we always compare the depth values of the pixels warped to the same coordinates, and the pixel with the closest range information to the camera always overwrites the other pixels. As the depth image is registered to the color image, the color pixels in $C_{a}$ can also be mapped along with the depth pixels to generate a virtual color image $C_{b}^{*}$ as well.

Then, the captured images $Z_{a}$, $C_{a}$, and virtual images $Z_{b}^{*}$, $C_{b}^{*}$ are decomposed into blocks of 8 × 8 pixels. In $Z_{b}^{*}$, some blocks have no depth information due to the fact that none of the pixels in $Z_{a}$ can be warped into these regions. This indicates that the blocks with the same coordinates in $Z_{b}$ and $C_{b}$ contain the information which can only be observed by sensor *b*. Sensor *a* collects these block coordinates in the set $B_{p}$ and transmits them to sensor *b*.

**Figure 5:**
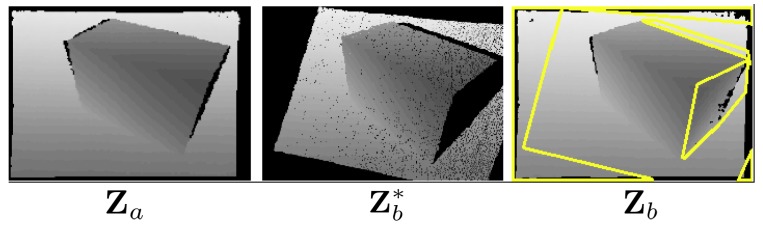
An intuitive example of the prediction process. The depth image $Z_{b}^{*}$ is synthetically generated from $Z_{a}$ as the image captured by sensor *b* virtually. The uncorrelated information in $Z_{b}$ is outlined with yellow lines.

An illustration of this process is shown in Figure 5. In this example, the regions in which the depth information can only be observed by $Z_{b}$ are outlined in yellow.

#### 3.3.2. Validation

**Figure 6:**
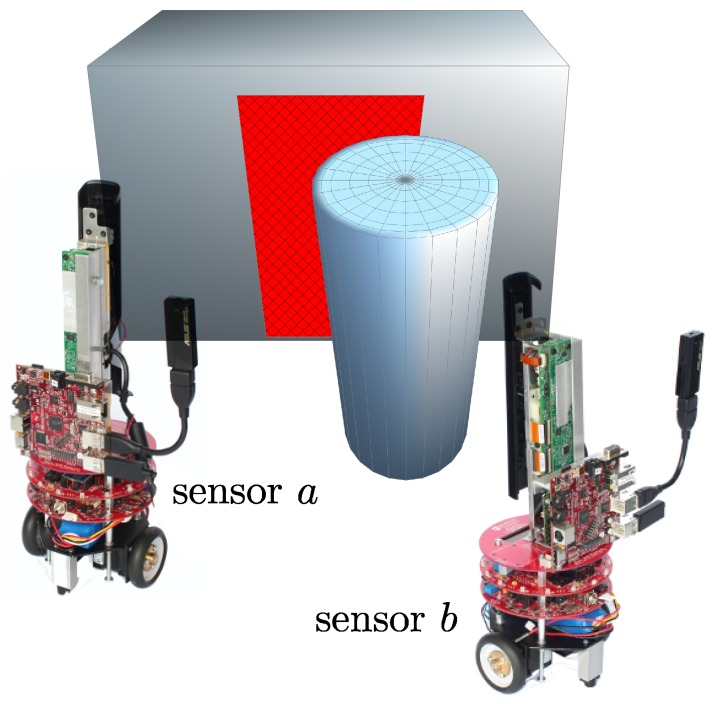
The rectangular surface area at the background is within the field of view of sensor *b*, but occluded by the cylinder at the foreground.

Although in most circumstances the prediction process can detect the uncorrelated information in the images captured by the other sensor, it may fail to operate correctly in situations when some points are occluded by the objects that can be seen by sensor *b*, but not by sensor *a*. A typical scenario is shown in Figure 6. In this example, the cylinder is outside the FoV of sensor *a*, and, because of this, it falsely treats some parts of the background (the dashed rectangular area) as the surface that is observable by sensor *b*. However, since the surface of the cylinder is included in $Z_{b}$, it occludes the background from the viewpoint of sensor *b*. As a result, the prediction process cannot accurately determine the uncorrelated depth and color information in this case.

In order to solve this problem, we include a validation mechanism into the overall process. First, similar to the image warping process from sensor *a* to *b*, sensor *b* generates the synthetic image $Z_{a}^{*}$ as virtually captured from sensor *a*’s viewpoint by mapping the pixels $\left(i_{b},j_{b}\right)$ in $Z_{b}$ to $\left(i_{a},j_{a}\right)$ in $Z_{a}^{*}$ by applying

$${\left[\begin{array}{cccc} \frac{i_{a}-i_{c}}{f_{x}} & \frac{j_{a}-j_{c}}{f_{y}} & 1 & \frac{1}{z_{a}} \end{array}\right]}^{T}\;=M_{ab}^{-1}\;{\left[\begin{array}{cccc} \frac{i_{b}-i_{c}}{f_{x}} & \frac{j_{b}-j_{c}}{f_{y}} & 1 & \frac{1}{z_{b}} \end{array}\right]}^{T}.$$

In this process, the pixels representing the range information of the surface of the cylinder move out of the image coordinate range and are not shown in $Z_{a}^{*}$. Sensor *b* identifies the image blocks containing these pixels, and records their coordinates in the set $B_{v}$. Then, sensor *b* transmits only the image blocks in $Z_{b}$ and $C_{b}$ that their coordinates are included in the union of the sets $B_{p}$ and $B_{v}$.

### 3.4. Image Coding

After the elimination of the redundant image blocks, the remaining uncorrelated depth and color information is compressed to further improve the communication channel usage.

For depth images, we use our own design *Differential Huffman Coding with Multiple Lookup Tables (DHC-M)* lossless compression scheme [21]. It is very fast and capable of compressing the depth images without introducing any artificial refinements.

Among the many options for compressing color images, JPEG 2000 [59] and H.264 [60] intra mode can be mentioned as the leading schemes. As the wireless channels are impacted by noise and being error prone, coding schemes that provide progressive coding are considered to be more suitable for sensor networks. Moreover, since a sensor node of a VSN has limited computational capability, a lightweight image coding scheme is required in sensor network applications. Progressive Graphics File (PGF) scheme [50], which is based on discrete wavelet transform with progressive coding features, has high coding efficiency and low complexity. It has compression efficiency comparable to JPEG 2000, and is ten times faster. Moreover, PGF has a small, open source and easy to use C++ codec [61] without any dependencies. These properties make PGF suitable for onboard image compression.

### 3.5. Post-Processing on the Decoder Side

On the decoder side (remote monitoring station), first, the received bitstream is decompressed. Then, the color and depth images captured by sensor *a* are used to predict the color and depth images captured by sensor *b*.

The 3D image warping process represented by Equation (1) may introduce some visual artifacts in the synthesized view, such as disocclusions (Disocclusions are areas occluded in the reference viewpoint and which become visible in the virtual viewpoint, due to the parallax effect.), cracks (Cracks are small disocclusions, and mostly occur due to undersampling.), or ghosts (Ghosts are artifacts due to the projection of pixels that have background depth and mixed foreground/background color.). Various methods have been proposed in the literature for their prevention or removal [62,63].

**Figure 7:**
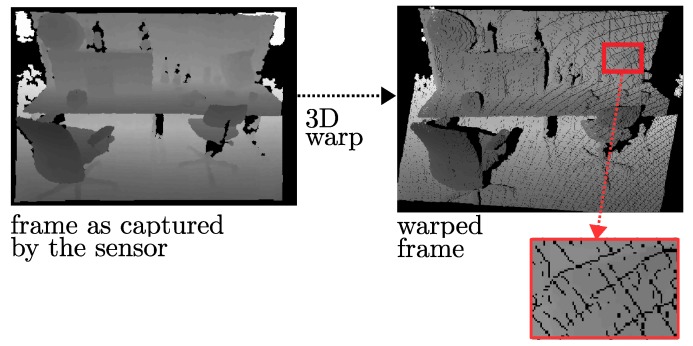
Crack artifacts: holes can be introduced during the image warping process due to the undersampling problem.

**Figure 8:**
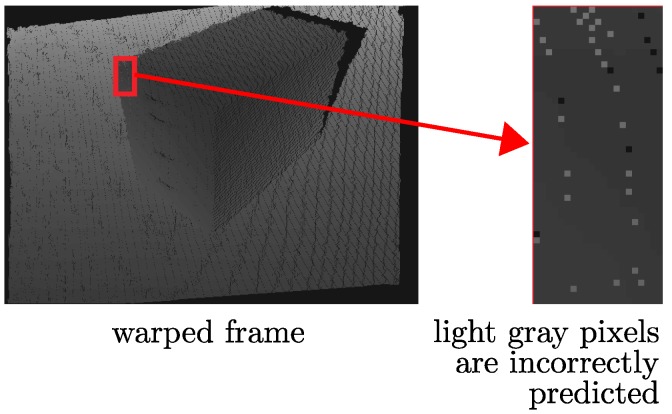
Ghost artifacts: the light gray pixels actually belong to the background surface and falsely warped onto the surface at the foreground.

In our framework, as the information that can only be observed by sensor *b* is transmitted, disocclusions can be eliminated by filling the areas affected by disocclusions in the synthesized image with the color and depth information transmitted by sensor *b*. Then, the main artifacts we need to deal with remain as cracks (Figure 7) and ghosts (Figure 8).

#### 3.5.1. Removal of Crack Artifacts

The missing color information in cracks is frequently avoided by operating a backward projection [64], which works in two steps:

- The cracks in the synthetic depth image are filled by a median filter, and then a bilateral filter is applied to smoothen the depth map while preserving the edges.
- The filtered depth image is warped back into the reference viewpoint to find the color of the synthetic view.

This approach exhibits good performance on filling the cracks, but at the same time it smoothens the complete image and introduces noise in regions with correct depth values, especially on the object boundaries. In order to avoid this adverse effect, we have modified it by using an adaptive median filter. The filter is applied only on the pixels with invalid depth values instead of the whole image. Instead of warping back the complete image to find the color information, we have adopted the work presented in [65], which warps back only the filled pixels in cracks, because the color information of the other pixels that are not in cracks can be directly estimated in the warping process.

#### 3.5.2. Removal of Ghost Artifacts

As illustrated in Figure 8, some background surfaces are incorrectly shown on the foreground obstacle’s surface. This is because the pixels representing the foreground surface become scattered after the warping process, and the background surface can be seen through the interspaces between these pixels. In order to remove this noise, we need to first identify the location of the incorrectly predicted pixels and then fill them with the correct values. As the value of the incorrectly predicted pixel is significantly different from its neighboring pixels, this kind of impulse noise can also be revised by using an adaptive median filter. We propose a windowing scheme with a 3×3 pixels size to determine whether or not a depth pixel contains incorrect values. If more than half of the neighboring pixels are out of a certain range, which is either much larger or much smaller than the center pixel in the window, the center pixel is estimated as an incorrectly predicted pixel. Then, it is replaced with the median value of its neighboring pixels, which are not out of the range. The corresponding color information can be found by backward warping, which is similar to the solution for crack artifacts presented in Section 3.5.1.

## 4. Experimental Results and Performance Evaluation

In this section, we first evaluate the performance of the relative pose estimation algorithm. Then, we analyze the overall performance of the RPRR framework through the experiments conducted on our mobile VSN platform.

### 4.1. Performance Evaluation of the Relative Pose Estimation

In order to quantitatively evaluate the performance of the relative pose estimation algorithm, we used two groups of datasets with varying degrees of occlusions. We first generated our own datasets by using a turntable setup to obtain the imagery viewed from accurately measured angular positions. A number of objects were placed on the center of the turntable, and the images were captured with a tripod mounted Kinect sensor [1]. In the experiments with the first dataset group, the ground truth is known exactly at every precisely controlled 5° interval. We used this setup to compare our algorithm (ICP-BD) [24] with the standard ICP [66] and ICP in inverse depth coordinates (ICP-IVD) [55]. The performance of the algorithms was evaluated based on the rotational and translational Root Mean Square (RMS) errors. The results show that

1. When the angular interval becomes greater than 15°, an increasing amount of occlusion occurs between two sensors’ views. Under such circumstances, ICP-BD outperforms other variants as it reports much lower translational and rotational RMS error.
2. Standard ICP has the poorest performance across the experiments. ICP-IVD can provide similar accuracy in pose estimation before it diverges. However, as the scene becomes more occluded as the turntable is being rotated, ICP-IVD fails to converge sooner than ICP-BD.

In summary, ICP-BD estimation accuracy is much better than that of ICP and ICP-IVD. In addition, its estimation is very robust even under large pose differences. Details of the experiment methodology and results can be found in [24].

**Figure 9:**
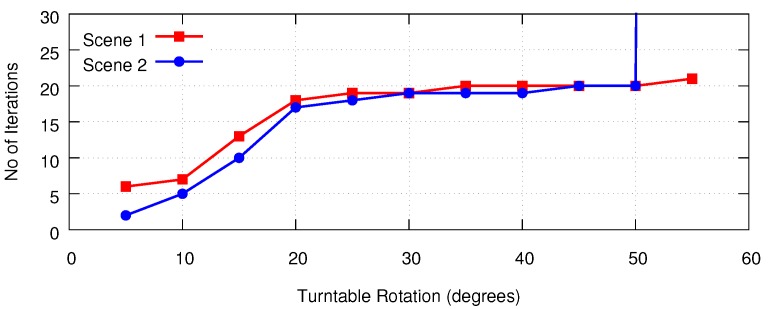
Number of iterations required by the ICP-BD algorithm in two scenes shown in Figure 5 of [24].

We also evaluated the number of iterations required for the ICP-BD algorithm’s convergence. Our experiments show that, as one can expect, the number of iterations increases as the angular difference between two views increases. Two representative results are plotted in Figure 9.

**Figure 10:**
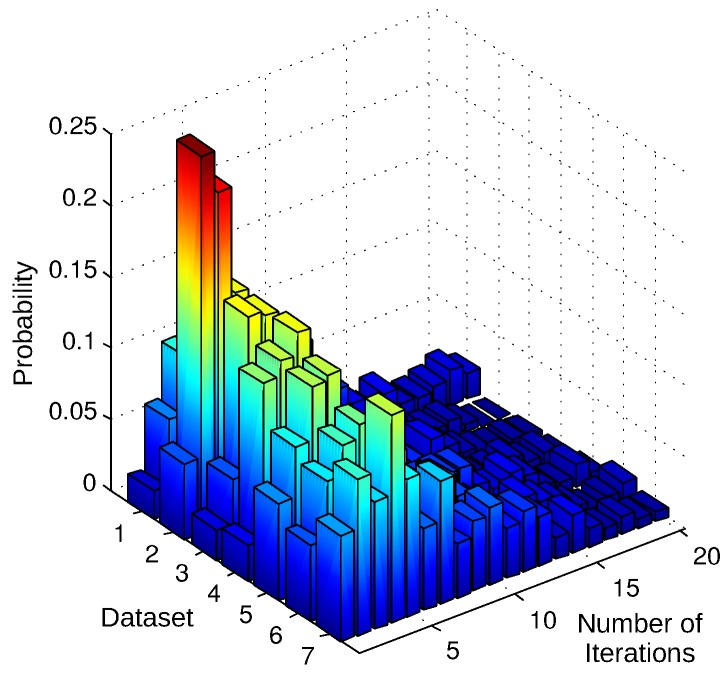
Distributions of the number of iterations required for the convergence of the algorithm. We experimentally obtained them over 4005 trials by using the 28 sequences we constructed by extracting image pairs that are 5, 10, 20 and 30 frames apart from the following seven RGB-D SLAM datasets [67,68]: 1. freiburg1_plant (1139 frames), 2. freiburg2_dishes (3005 frames), 3. freiburg3_cabinet (1121 frames), 4. freiburg3_large_cabinet (993 frames), 5. freiburg3_structure_texture_far (914 frames), 6. freiburg3_long_office_household (2509 frames), and 7. freiburg1_xyz_cabinet (800 frames). The plot shows that, for example, the algorithm converged after three iterations in 23% of the trials in the four sequences extracted from the dataset 2.

In order to gain further insight into the number of iterations required by our algorithm in densely cluttered scenes, we used a second group of datasets which were selected from the Technical University of Munich Computer Vision Group’s RGB-D SLAM dataset and benchmark collection [67,68]. Each dataset is a sequence of Kinect video frames capturing one scene from different angles of view. For emulating the situations including varying amounts of occlusion between two sensor views, we created four new sequences from each dataset by extracting one frame out of every 5, 10, 20, and 30 frames. For each trial, we treated two consecutive frames in these 28 new sequences as the depth images captured by two separate sensors with varying relative poses. We recorded the number of iterations required by the ICP-BD algorithm that converged successfully. We normalized the results of 4005 trials to plot them as discrete probability distributions as shown in Figure 10. The results show that the average number of iterations is 5.1 and the maximum value is smaller than 20. Based on these numbers, we can say that the message exchange complexity of the relative pose estimation algorithm is near-constant. At each iteration, the depth information and image coordinates of 250 sampled points need to be transmitted, which lead to 1.09 kB of bandwidth consumption approximately (excluding the protocol overheads). Therefore, on average, 5.6 kB of data are sent in each message when the relative localization algorithm (Figure 4) distributed over sensors *a* and *b* is in operation.

### 4.2. Performance Evaluation of the RPRR Framework

**Figure 11:**
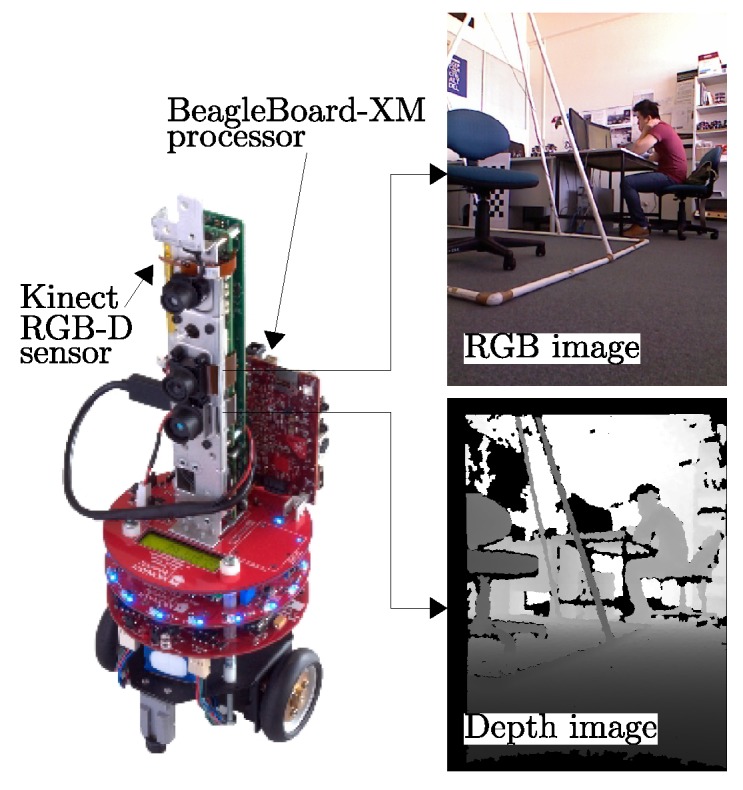
eyeBug [69,70], the mobile RGB-D sensor we used in our experiments. The color and depth data generated by the Kinect sensor is processed on a BeagleBoard-xM [71] computer running the GNU/Linux operating system.

In this set of experiments, we evaluated the performance of the RPRR framework by using two mobile RGB-D sensors (Figure 11) of our VSN platform. The platform consists of multiple mobile RGB-D sensors named “eyeBug” (Figure 11). EyeBugs were designed for computer vision and mobile robotics experiments, such as multi-robot SLAM and scene reconstruction. We selected the Microsoft Kinect as the RGB-D sensor due to its low cost and wide availability. We mounted a Kinect vertically at the center of the top board of each eyeBug.

A Kinect is capable of producing color and disparity-based depth images at a rate of 30 frames/second. A BeagleBoard-xM single-board computer [71] was used for image processing tasks. Each BeagleBoard-xM has a 1 GHz ARM Cortex-A8 processor, a USB hub, and an HDMI video output port. A USB WiFi adapter was connected to the BeagleBoard to provide communication between robots. We ran an ARM-processor-optimized Linux kernel. OpenKinect [72], OpenCV [73] and libCVD [74] libraries were installed to capture and process image information. The default RGB video stream provided by the Kinect uses eight bits for each color at VGA resolution (640 × 480 pixels, 24 bits/pixel). The monochrome depth video stream is also in VGA resolution. The value of each depth pixel represents the distance information in millimeters. Invalid depth pixel values are recorded as zero, indicating that the RGB-D sensor is not able to estimate the depth information of that point in the 3D world.

**Figure 12:**
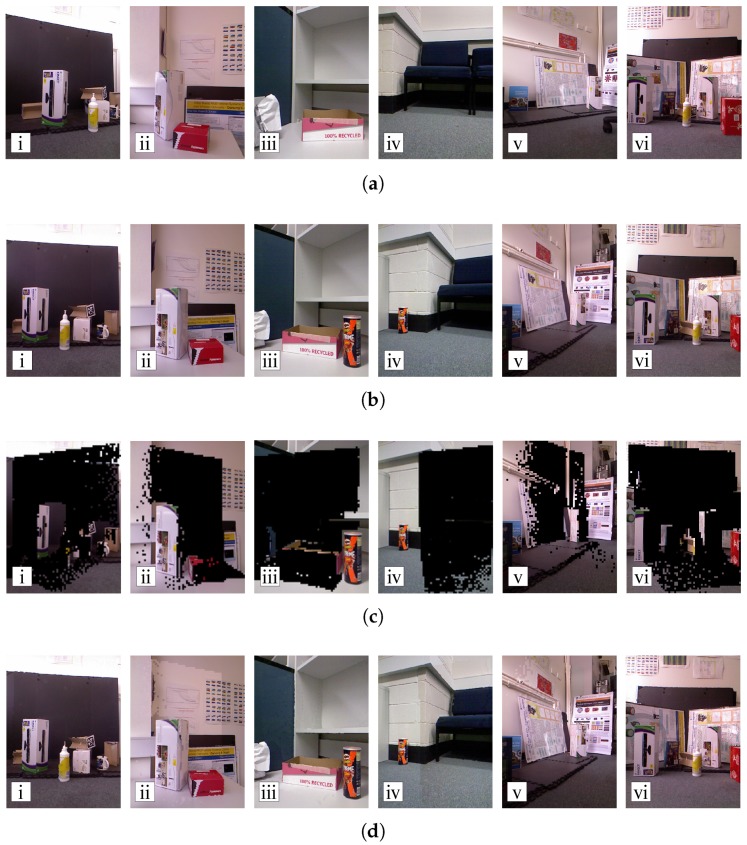
A demonstration of the RPRR framework over six scenes. (**a**) images captured and transmitted to the remote monitoring station by sensor *a*; (**b**) images captured by sensor *b*. Note that these images are not transmitted to the remote monitoring station; (**c**) image blocks transmitted by sensor *b* (black regions denote the parts of an image that are identified as redundant by our scheme and consequently not transmitted); (**d**) reconstructed images of sensor *b*’s point of view. They are produced at the remote monitoring station using the partial images transmitted by sensor *b* shown in row (**c**), and ideally should be identical to the corresponding images in row (**b**).

Color and depth images were captured in six different scenes, as shown in Figure 12. In this set-up, sensor *a* transmits entire captured color and depth images to the central monitoring station. Then, sensor *b* is required to transmit only the uncorrelated color and depth information that cannot be observed by sensor *a*. At the central monitoring station, the color and depth images captured by sensor *b* are reconstructed by using the information transmitted by two sensors.

As the color and depth images captured by sensor *a* are compressed and transmitted to the receiver in their entirety, we only needed to evaluate the reconstruction quality of the images captured by sensor *b*. The depth images are usually complementary to the color images in many applications, and in our framework the color images are reconstructed according to depth image warping. Thus, if the color images can be accurately reconstructed, so the reconstructed depth images as well. Therefore, in this set of experiments, we focused on evaluating the quality of the reconstructed color images.

#### 4.2.1. Subjective Evaluation

The image blocks transmitted by sensor *b* are shown in the third row of Figure 12. In the fourth row of the figure, reconstructed images can be seen. They were obtained by stitching the blocks extracted from the warped sensor *a* images into the black regions of the corresponding sensor *b* images. In the reconstructed images of scenes 2 and 4, we observe significant color changes on the stitching boundary. This is caused by the illumination variations within the scene, and auto-iris response of the sensors to different levels of scene brightness.

Generally, it is clear that the reconstructed images preserve the structural information of the original images accurately.

#### 4.2.2. Objective Evaluation

Even though many approaches have been proposed to compress multi-view images [17,34,36,42,75,76,77], they cannot be applied in our system. These approaches either require the transmitter to have the knowledge of the full set of images or only work on cameras with very small motion differences. In contrast, in our case, each sensor only has its own captured image, and the motion difference between two visual sensors is very large. To the best of our knowledge, our proposal is the first distributed framework that efficiently codes and transmits images captured by multiple RGB-D sensors with large pose differences, and so we do not have any work to compare ours against. For this reason, we can only compare the performance of our framework with the approaches that compress and transmit images independently.

**Figure 13:**
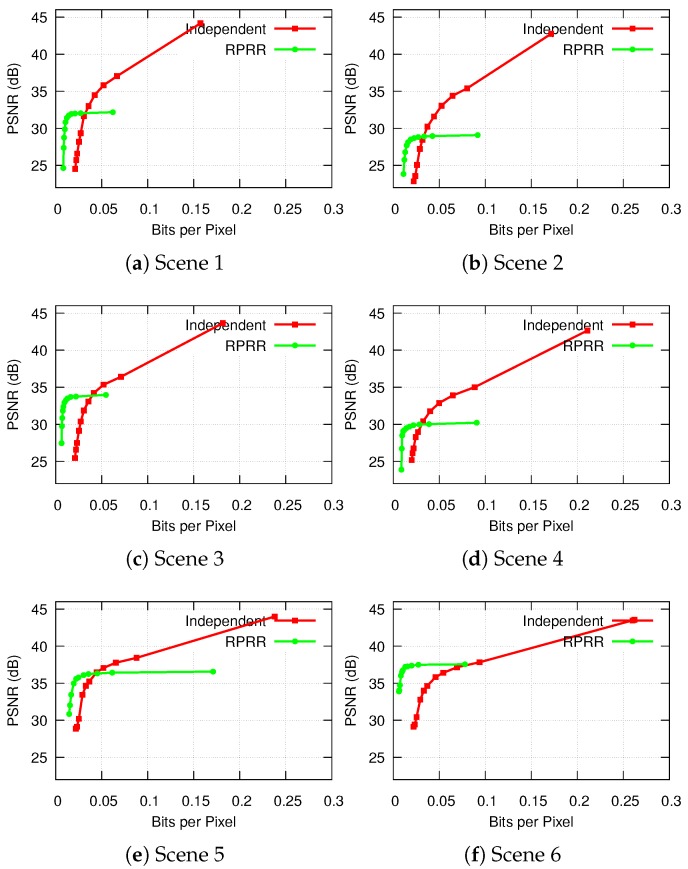
Comparisons of PSNR (dB) achieved by compressing the images at various levels by using the RPRR framework against transmitting them independently.

As the color information is coded using the PGF [50] lossy mode, we can vary the compression ratio, and, consequently, coding performance. The performance was evaluated according to two aspects: reconstruction quality and bits per pixel (bpp). We measured the Peak-Signal-to-Noise-Ratio (PSNR) between the reconstructed and original images captured by sensor *b* with different bpp. The results are shown in Figure 13.

Figure 13 shows that the RPRR framework can achieve much lower bpp than the independent transmission scheme. However, the PSNR upper bounds achieved by the RPRR framework are limited. It is because the reconstruction quality depends on the depth image accuracy and correlations between the color images. Since the depth images generated by a Kinect sensor are not accurate enough, the displacement distortion of depth images, especially the misalignment around the object edges, introduces noise into the reconstruction process. Another reason is the inconsistent illumination between the color images captured by two sensors. Even if the prediction and validation processes establish the correct correspondences between two color pixels according to the transformation between depth images, the values of these two color pixels can be very different due to the various brightness levels in two images. These characteristics lead to low PSNR upper bounds of the reconstructed color images. Several methods [78,79] have been proposed to overcome this drawback; however, the time-complexity of these methods prevents them from being implemented on sensor systems with constrained computational resources. We can see that the reconstructed color image in Scene 6 has the highest PSNR. This is because the relative pose between two sensors is small, which leads to small differences in the structure of the captured scenes and the brightness of their captured images. Therefore, more information captured by sensor *b* can be reconstructed by information observed by sensor *a*. For that reason, according to Figure 12(a-vi), only a small number of blocks in images captured by sensor *b* need to be transmitted. We also observe that Scenes 2 and 4 have the lowest reconstruction qualities. This is because the brightness level is quite different in the color images captured by two sensors (see image pairs shown in Figure 12(a-ii,b-ii) and Figure 12(a-iv,b-iv)). Although the structures of the scenes are preserved nicely in the reconstructed color images, distinct color changes over the stitching boundaries are shown in Figure 12(d-ii,d-iv). Consequently, we can say that the RPRR framework is suitable for implementation of the VSN applications with very limited bandwidth requiring very high compression ratios. This is because when the bpp or the compression ratio increases, the quality of the color image reconstructed by RPRR decreases more gradually than the quality of the image compressed by the independent transmission scheme.

#### 4.2.3. Energy Consumption

The limited battery capacity of mobile sensors places limits on their performance. Therefore, a data transmission scheme, while attempting to reduce the transmission load, must not have a significant negative impact on the overall energy consumption. In this section, we present our experimental measurements and evaluation regarding the overall energy consumption and amount of transmitted data of the RPRR framework collected on our eyeBug mobile visual sensors to demonstrate this aspect.

The overall energy consumption of the RPRR framework can be measured by

(16) $$\begin{array}{cc} E_{\mathrm{overall}}^{R} & =E_{\mathrm{processing}}+E_{\mathrm{encoding}}+E_{\mathrm{sending}}\\ & =V_{o}I_{p}t_{p}+V_{o}I_{e}t_{e}+V_{o}I_{s}t_{s} \end{array}$$

in which $V_{o}$ denotes the sensor’s operating voltage, and $I_{p},I_{e},$ and $I_{s}$ represent the current drawn from the battery during processing, encoding, and sending operations. $t_{p},t_{e},$ and $t_{s}$ are the corresponding operation times required for these procedures.

The overall energy consumption when images are transmitted independently can be measured as

(17) $$\begin{array}{cc} E_{\mathrm{overall}}^{I} & =E_{\mathrm{encoding}}+E_{\mathrm{sending}}\\ & =V_{o}I_{e}t_{e}+V_{o}I_{s}t_{s}. \end{array}$$

Note that the operation times $t_{e}$ and $t_{s}$ are different in the two transmission schemes as the image sizes change after removing the redundant information.

Our sensor operates at 15 V, and the current levels remain fairly constant during each operation. We measured them as follows: $I_{p}=0.06\;A$, $I_{e}=0.06\;A$, and $I_{s}=0.12\;A$. Our experiments show that, in the RPRR framework, due to different compression ratios, the transmission time varies between 32 and 42 ms, and the operational time for processing and encoding remains between 509 and 553 ms. The overall energy consumption of the RPRR scheme changes between 480 and 520 mJ, depending on the compression ratio. The corresponding values for the independent scheme are between 918 and 920 mJ. The data clearly show that the RPRR framework leads to the consumption of much lower battery capacity than the independent transmission scheme. It cuts the overall energy consumption of the sensor nearly by half. In the RPRR framework, the energy consumption for two sensors are asymmetric, and if sensor *a* always transmits complete images, its energy will be quickly drained. A simple method to prolong the network lifetime is for the two sensors to transmit complete images alternately. The current consumed by an eyeBug in idle state is 650 mA. According to the experimental results above, the theoretical operational time of RPRR on a pair of eyeBugs with 2500 mAh 3-cell (11.1 V) LiPo batteries is around 5.2 h. In this period, around 3.24 × 10^4^ color and depth image pairs can be transmitted to the remote monitoring station.

#### 4.2.4. Transmitted Data Volume

**Figure 14:**
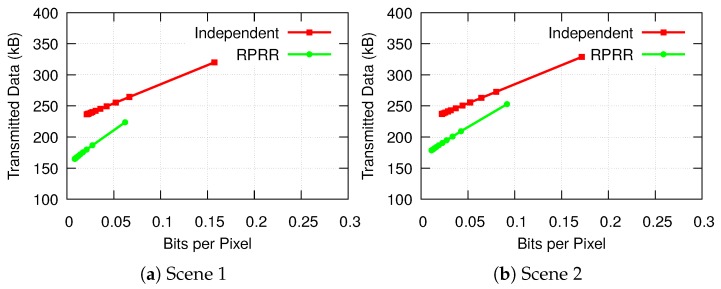

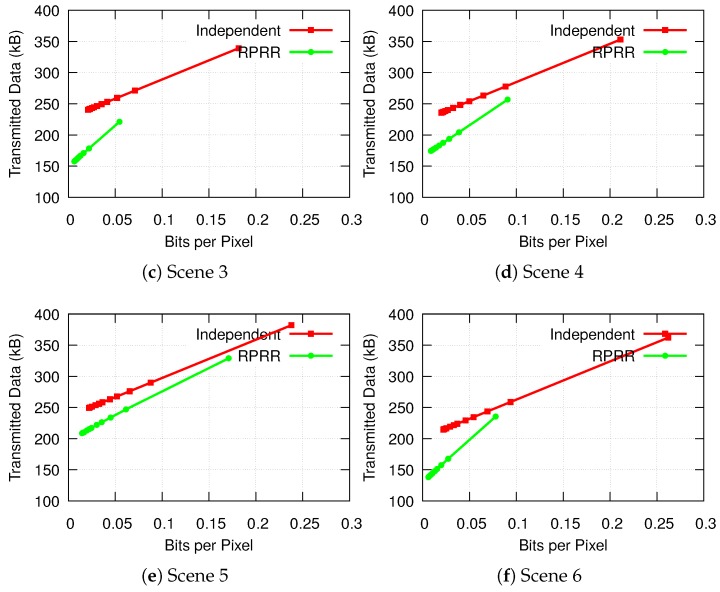
Comparisons of the transmitted data for color images at various compression levels by using the RPRR framework against transmitting them independently.

Finally, we compare the amount of transmitted data for two pairs of color and depth images required by the RPRR and the independent transmission schemes. The results are shown in Figure 14. We can see that bits per pixel achieved by the independent transmission approach is much higher than bit per pixel achieved by the RPRR framework. It is also noticeable that even if the bits per pixel required by a color image is the same in both approaches, the RPRR framework transmits fewer number of bytes. This is because only parts of the color and depth images need to be transmitted in RPRR. In contrast, a complete depth image has to be sent in the independent transmission scheme. The data clearly show that the RPRR framework leads to more efficient use of the wireless channel capacity than the independent transmission scheme.

## 5. Conclusions

We presented a novel collaborative transmission framework for mobile VSNs that efficiently removes the redundant visual information captured by RGB-D sensors. The scheme, called *Relative Posed based Redundancy Removal* (RPRR), considers a multiview scenario in which pairs of sensors observe the same scene from different viewpoints. Taking advantage of the unique characteristics of depth images, our framework explores the correlation between the images captured by these sensors using solely the relative pose information. Then, only the uncorrelated information is transmitted. This significantly reduces the amount of information transmitted compared with sending two individual images independently. The scheme’s computational resource requirements are quite modest, and it can run on battery-operated sensor nodes. Experimental results show that the compression ratio achieved by the RPRR framework is 2.5 times better than the independent transmission scheme, and it yields this result while nearly halving the energy consumption of the independent transmission scheme on average.

The RPRR framework is the first attempt to remove the redundancy in the color and depth information observed by VSNs equipped with RGB-D sensors, and so there is room for further improvements. For example, our scheme only operates on pairs of mobile sensors at this stage. A simple extension of the RPRR framework for networks with a large number of RGB-D sensors is to choose one sensor as the reference which transmits complete images (like sensor *a* in Figure 2) while the other sensors transmit only the uncorrelated information (like sensor *b* in Figure 2). However, a certain amount of redundancy still exists in this approach and further refinements are possible.

Our future research efforts will concentrate on developing a more sophisticated extension which uses feature matching algorithms to assign sensors with overlapping FoVs to the same subgroups and applies RPRR on sensors in the same subgroup to remove redundancies in networks with a large number of RGB-D sensors.
